# Characterization of the EM66 Biomarker in the Pituitary and Plasma of Healthy Subjects With Different Gonadotroph Status and Patients With Gonadotroph Tumor

**DOI:** 10.3389/fendo.2019.00102

**Published:** 2019-02-22

**Authors:** Johann Guillemot, Marlène Guérin, Anne-Françoise Cailleux, Antoine-Guy Lopez, Jean-Marc Kuhn, Youssef Anouar, Laurent Yon

**Affiliations:** ^1^Laboratory of Neuronal and Neuroendocrine Differentiation and Communication, Normandie Univ, UNIROUEN, INSERM, Rouen, France; ^2^Endocrinology, Diabetes and Metabolism Department, Normandie Univ, UNIROUEN, Rouen University Hospital, INSERM CIC-CRB, Rouen, France; ^3^Department of Endocrinology, Diabetes and Metabolic Diseases, Normandie Univ, UNIROUEN, Rouen University Hospital, Rouen, France

**Keywords:** EM66, pituitary, gonadotroph tumor, plasma marker, secretogranin II, neuroendocrine tumors

## Abstract

Granins and their derived-peptides are useful markers of secretion from normal and tumoral neuroendocrine cells. The need to identify new diagnostic markers for neuroendocrine tumors, including pituitary tumors prompted us to determine plasma levels of the secretogranin II-derived peptide EM66 in healthy volunteers with different gonadotroph status and to evaluate its usefulness as a circulating marker for the diagnosis of gonadotroph tumor. Using a radioimmunoassay, we determined plasma EM66 concentrations in healthy men and women volunteers in different physiological conditions in relation with the gonadotroph function. Our results revealed that in men, in women with or without contraception, in pregnant or post-menopausal women, plasma EM66 concentrations are not significantly different, and did not show any correlation with gonadotropin levels. In addition, stimulation or inhibition tests of the gonadotroph axis had no effect on EM66 levels, whatever the group of healthy volunteers investigated while gonadotropin levels showed the expected variations. Immunohistochemical experiments and HPLC analysis showed the occurrence of EM66 in pituitary gonadotroph, lactotroph and corticotroph tumors but not in somatotroph tumor. In patients with gonadotroph or lactotroph tumor, plasma EM66 levels were 1.48 (0.82–4.38) ng/ml and 2.49 (1.19–3.54) ng/ml, respectively. While median value of EM66 was significantly lower in patients with gonadotroph tumor compared to healthy volunteers [2.59 (0.62–4.95) ng/ml], plasma EM66 concentrations were in the same range as normal values and did not show any correlation with gonadotropin levels. These results show that plasma EM66 levels are independent of the activity of the gonadotroph axis in healthy volunteers and, while EM66 levels are reduced in gonadotroph tumors, plasma EM66 does not provide a helpful marker for the diagnosis of these tumors.

## Introduction

EM66 is a 66 amino acid peptide generated from secretogranin II (SgII) by cleavage at dibasic amino acid sites (1). EM66 has been identified in adult and fetal human adrenal gland (1) and subsequently in rat and bovine adrenochromaffin cells (2, 3). In rodents, EM66 immunoreactivity was also found in several hypothalamic regions (4, 5), which suggests several neuroendocrine roles for EM66 as it was proposed recently for the control of feeding behavior (6, 7). Moreover, we have previously demonstrated that measurement of tissue concentrations of EM66 may help to discriminate between benign and malignant pheochromocytomas (8–10) and that EM66 is secreted from pheochromocytoma tissue (11) and represents a sensitive plasma marker that should be considered as a complementary tool for the diagnosis and follow-up of pheochromocytoma (12–14).

SgII is a major product of gonadotrophs (15) and EM66-immunoreactivity has been found in gonadotroph cells of the rat pituitary (2). Moreover, high SgII mRNA levels have been detected in human pituitary gonadotroph adenomas (16) whereas in pheochromocytomas, SgII mRNA, and plasma EM66 levels are significantly correlated (10, 17). Together, these data suggest that EM66 could be a marker for pituitary gonadotroph tumors. As recently recommended by the International Pituitary Pathology Club (18, 19), we have decided to use the term of pituitary neuroendocrine tumor or pituitary tumor instead of pituitary adenoma.

In the present study, we report on a clinical trial performed first to determine plasma EM66 levels in healthy volunteers in different physiological conditions related to gonadotroph function. We also investigated the potential usefulness of EM66 as a marker for the diagnosis of pituitary gonadotroph tumors.

## Materials and Methods

### Subjects and Plasma Samples

Plasma samples were collected from 40 healthy volunteers and 28 patients with pituitary tumor. They were provided by the Rouen University Hospital, INSERM CIC-CRB 1404 within the context of clinical trials (n° 0317HP for healthy men, n° 03158HP for healthy women, and n° 03159HP for patients with pituitary tumors). Control subjects were divided into 5 groups (*n* = 8 each): group I, men; group II, women without contraception; group III, women with anti-oestro-progestative contraception; group IV, postmenopausal women without hormone replacement therapy, and group V, pregnant women. Fourteen patients (7 men 58.7 ± 10.3 years, 7 women 53.9 ± 20.9 years) with gonadotroph tumor and 14 patients (4 men 37 ± 19.2 years, 10 women 36.2 ± 18.1 years) with lactotroph tumor were also included in this study. Control subjects and patients gave written informed consent, and the protocol of collection of the samples was approved by the regional bioethic committee of Haute-Normandie. After collection, plasma samples were kept frozen at −80°C.

### Clinical Trial Protocols

All trials were performed at Rouen University Hospital, INSERM CIC-CRB 1404. Plasma EM66 concentrations were measured in basal and under stimulatory or inhibitory conditions of the gonadotroph axis in healthy men and women volunteer groups, as previously described (20). They were taking no medication, did not have any history of disease, and their clinical examination was normal.

#### Group I: Healthy Men Volunteers

A group of 8 volunteers was studied (18–60 years old). The first day of the study (D1, 08h00), blood samples were first collected in basal resting conditions. Then, 1 ml of vehicle followed by 100 μg of GnRH were injected intravenously and, for each test, blood samples were collected at time intervals of 15, 30, and 60 min, in order to measure the levels of EM66, LH, FSH, and the free alpha-subunit (FAS) of glycoprotein hormones in placebo or a stimulatory condition of the gonadotroph axis. Then, a treatment with percutaneous dihydrotestosterone (DHT, 125 mg twice a day) was initiated and maintained for 7 days. On D7, corresponding to an inhibitory condition of the gonadotroph axis, blood samples were collected using the same procedure as on D1.

#### Group II: Healthy Women Volunteers Without Contraception

A group of 8 volunteers (18–40 years old) in period of fertility and not under oestro-progestative contraception was studied. On D1 corresponding to the early follicular phase of the menstrual cycle, blood samples were collected as described for group I on D1 (basal, and GnRH-stimulated conditions). On D14 and on D22, corresponding respectively to the ovulation peak and the luteal phase (gonadotroph axis stimulatory conditions), a single blood sample was collected.

#### Group III: Healthy Women Volunteers With Oestro-Progestative Contraception

A group of 8 volunteers (18–40 years old) in period of fertility and under oestro-progestative contraception for at least 3 months was studied. On D1, corresponding to the few days of contraceptive treatment stop during the menstrual cycle, blood samples were withdrawn as described for group I on D1. On D25, corresponding to 25 days of contraceptive treatment (gonadotroph axis inhibitory condition), a single blood sample was collected.

#### Group IV: Post-Menopausal Healthy Women Volunteer

A group of 8 postmenopausal healthy volunteers (50–65 years old) without hormonal substitution treatment was studied. Blood samples were withdrawn as described for group I on D1 (basal and GnRH-stimulated conditions).

#### Group V: Pregnant Healthy Volunteers

A group of 8 healthy volunteers in the first quarter of pregnancy (18–35 years old) was studied. Through their medical monitoring program, a single blood sample was collected to measure EM66 and hormones concentrations.

### Immunohistochemical Procedure

Pituitary tumor slices were provided by the Rouen University Hospital, Endocrinology, Diabetes, and Metabolism Department. They were paraffined and fixed with formalin. After deparaffinizing and rehydration, tissues were processed for indirect immunofluorescence. Tissue sections of gonadotroph tumors were incubated overnight at 4°C simultaneously with two primary antibodies for double-labeling experiments, i.e., EM66 antiserum [code number 736–1806; (1)] diluted 1:1,000 or monoclonal LHß antibody diluted 1:300 in PBS containing 0.3% Triton X-100 and 1% bovine serum albumin (BSA; Roche Diagnostics, Mannheim, Germany). Tissue sections were then rinsed in PBS and incubated at room temperature for 90 min with the appropriate secondary antibody, i.e., Goat Anti-Rabbit/Alexa-488 diluted 1:300, Donkey Anti-Sheep/Alexa-488 diluted 1:300, Goat Anti-Mouse/Alexa-594 diluted 1:300, or Donkey Anti-Rabbit/Alexa-594 diluted 1:300. Nucleic acids in the cell nuclei were counterstained with 4,6-diaminodo-2-phenylindole (DAPI). Finally, slices were rinsed in PBS, mounted in PBS/glycerol (1:1), cover-slipped, and examined using a confocal laser scanning microscope (CLSM TCS-SP2-AOBS, Leica) equipped with a fluorescence DMRX-A2 microscope and an argon (excitation wavelengths 458/476/488/514 nm) and two helium/neon (excitation wavelengths 543 and 633 nm, respectively) ion lasers. To verify the specificity of the immunoreaction, the following controls were performed: (1) substitution of the primary antibodies with PBS, (2) incubation with non-immune serum instead of the primary antisera, and (3) preincubation of the EM66 antiserum (diluted 1:1,000) with the corresponding synthetic peptide (10^−6^ M).

Given that the EM66 and PRL antisera were raised in rabbit, the following procedure was performed as previously described (21). Tissue sections of lactotroph tumors were incubated overnight at 4°C with the EM66 antiserum diluted 1:1,000. Then they were rinsed in PBS and incubated at room temperature for 90 min with GAR/Alexa-488 diluted 1:300. After rinsing, slices were post-fixed in 2% paraformaldehyde in PBS to allow fixation of the EM66-GAR immunoreactive complexes. Subsequently, they were incubated overnight at 4°C with the PRL [code 19602; (22)] antiserum diluted 1:500 and then incubated for 90 min at room temperature with GAR/Alexa-594 diluted 1:300. To verify the specificity of the immunoreaction and the performance of the post-fixation procedure, the following controls were performed: (1) substitution of the primary antibodies with PBS, (2) incubation with non-immune rabbit serum instead of the primary antisera, (3) preincubation of the EM66 antiserum (diluted 1:1,000) with the corresponding synthetic peptide (10^−6^ M), (4) substitution of the secondary antibodies with PBS.

Immunoenzymatic labeling of gonadotroph tumor or normal pituitary tissue slices was performed using a commercial kit (EnVision+ System, Peroxidase; DAKO Corp., Carpinteria, CA). The tissue slices were incubated for 5 min with a 0.03% hydrogen peroxide solution to quench any endogenous peroxidase activity. After several rinses in distilled water, the slides were incubated for 30 min with the EM66 antiserum diluted 1:5,000 in Tris-buffered saline (0.05 m Tris-HCl; 0.15 m NaCl, pH 7.4; TBS) containing 0.3% Triton X-100 and 1% BSA. Slices were rinsed in TBS and then incubated for another 30 min with a peroxidase-labeled polymer conjugated to goat antirabbit γ-globulins. Finally, a 3, 3′-diaminobenzidine chromogen solution was applied for 2–3 min, the tissues were rinsed in distilled water, counterstained with hematoxylin, and mounted with Eukitt medium. To verify the specificity of the immunoreaction, the following controls were performed: (1) substitution of the primary antibodies with PBS, (2) incubation with non-immune serum instead of the primary antisera, and (3) preincubation of the EM66 antiserum (diluted 1:1,000) with the corresponding synthetic peptide (10^−6^ M).

### HPLC Analysis and RIA of EM66

The HPLC analysis and RIA procedure were performed as previously reported (1). The plasma EM66 concentrations were measured by RIA as previously described (12). Assay precision of EM66 has been previously reported (12).

### Hormone Asays

LH and FSH were measured by their respective chemiluminescent assays (Immulite 2500, Siemens Diagnostics, La Garenne Colombe, France). Plasma FAS levels were measured by the previously described RIA (23).

### Data Analysis

Data are reported as median (min–max) or mean ± SEM. Several non-parametric statistical methods were used, Mann–Whitney *U*-test and Kruskal–Wallis test. The Spearman's test was performed to analyze the correlations between plasma levels of EM66 and gonadotropins. Probability values < 0.05 were considered significant. Data were analyzed with the Prism program (GraphPad Software, San Diego, CA).

## Results

### Basal EM66 Levels in Plasma of Healthy Volunteers

**Figure 1:**
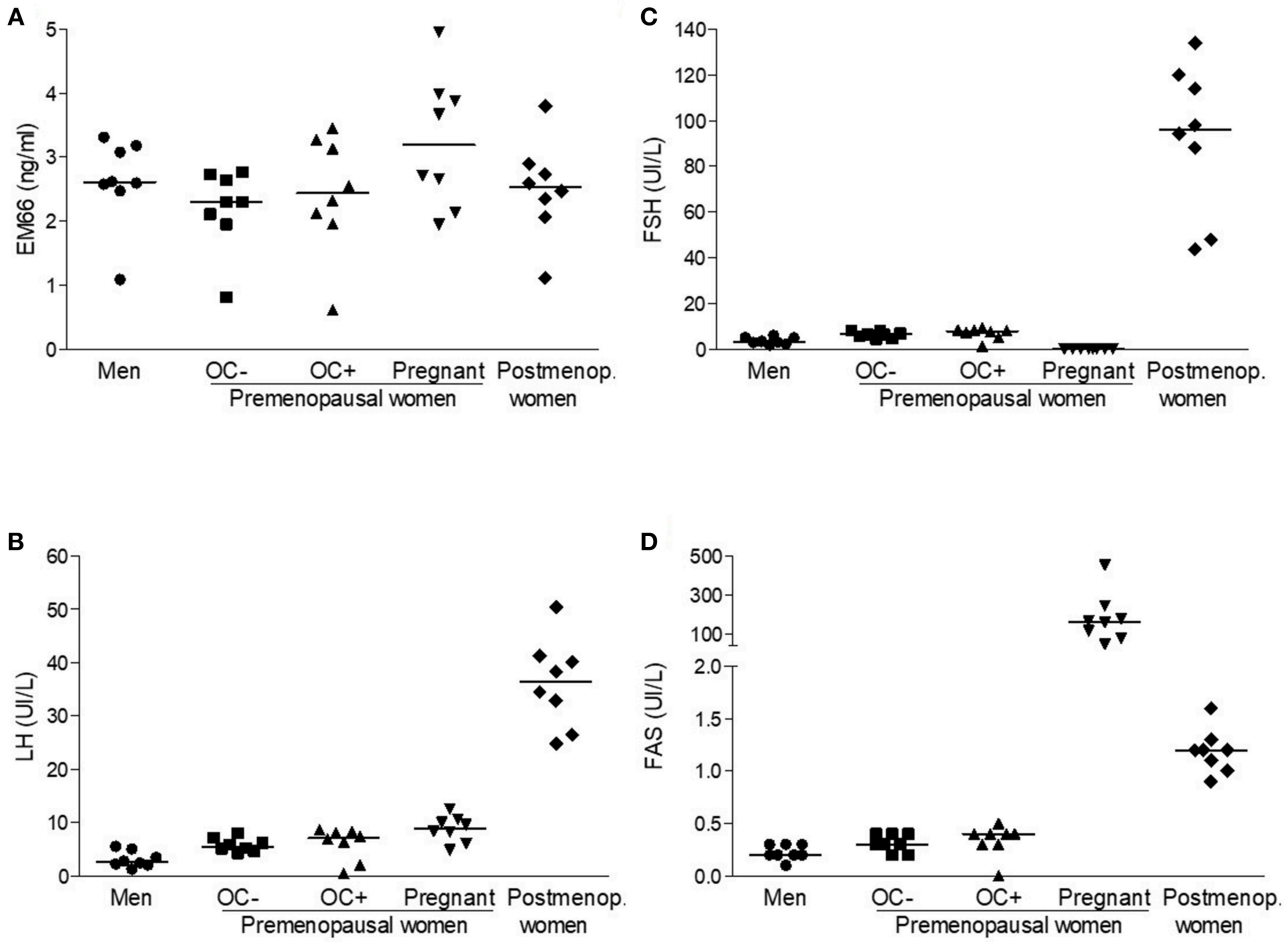
EM66 and gonadotropin levels in plasma of healthy volunteers. Scattergram of plasma EM66 **(A)**, LH **(B)**, FSH **(C)**, and FAS **(D)** concentrations in men (•), in premenopausal women without (■, OC-) or with (▴, OC+) oral contraception (OC), in pregnant (▾) or postmenopausal without hormone replacement therapy (postmenop., ♦) women. The *bars* represent the median value for each group.

Plasma EM66 concentrations in healthy volunteers were 2.61 (1.09–3.31) ng/ml (*n* = 8, Figure 1A) in men, 2.30 (0.81–2.76) ng/ml (*n* = 8) in premenopausal women without oral contraception, 2.44 (0.62–3.45) ng/ml (*n* = 8) in premenopausal women with oral contraception, 3.19 (1.94–4.95) ng/ml (*n* = 8) in pregnant women and 2.53 (1.11–3.80) ng/ml (*n* = 8) in postmenopausal women (Figure 1A). Plasma EM66 concentrations were not significantly different between the different groups and were in the same range as those previously reported in other human plasma samples (12, 14). Finally, no correlation was found for any group between plasma EM66 levels and plasma LH (Figure 1B), FSH (Figure 1C) or FAS (Figure 1D) levels.

### Plasma EM66 Levels of Healthy Volunteers During Stimulatory or Inhibitory Conditions of the Reproductive Axis

**Figure 2:**
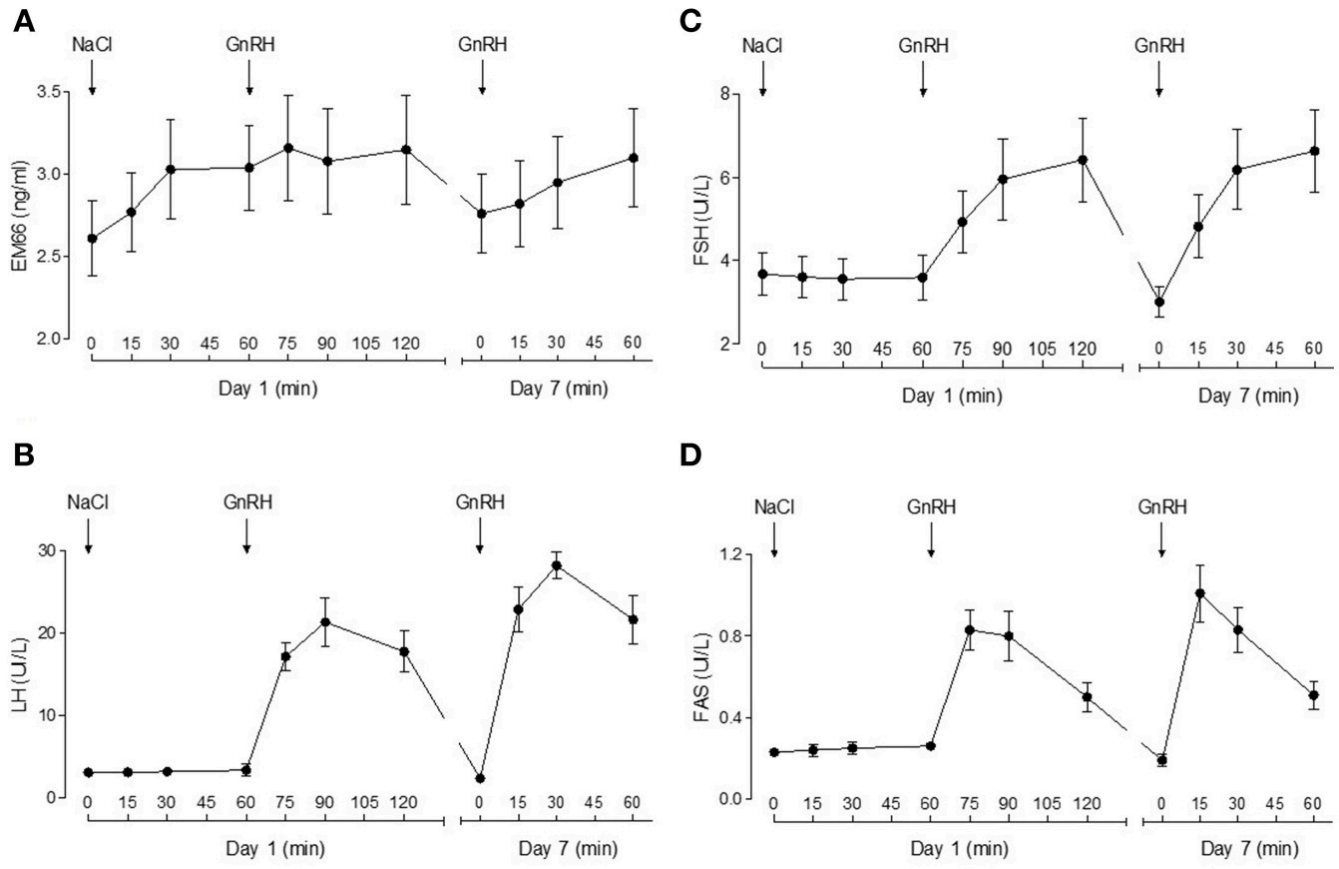
Plasma EM66 and gonadotropin levels of healthy men during stimulatory or inhibitory conditions of the reproductive axis. Results of dynamic tests for evaluation of EM66 **(A)**, LH **(B)**, FSH **(C)**, and FAS **(D)** levels in plasma of healthy men. Day 1: response of EM66 and gonadotropins after intravenous administration of vehicle followed by 100 μg GnRH. Day 7: response of EM66 and gonadotropins after 7 days administration of percutaneous DHT (125 mg twice daily) followed by 100 μg GnRH. Values are means ± SEM (*n* = 8).

On day 1, plasma EM66 concentrations did not change significantly after vehicle or GnRH treatment in men (Figure 2A). On day 7 of the treatment with DHT whose effectiveness was confirmed by the decrease in plasma testosterone levels, plasma EM66 levels were similar to those obtained at the first day of the test and were not significantly modified by the addition of GnRH (Figure 2A). In contrast, plasma LH (Figure 2B), FSH (Figure 2C), and FAS (Figure 2D) levels were increased in response to GnRH treatment as expected (20).

**Figure 3:**
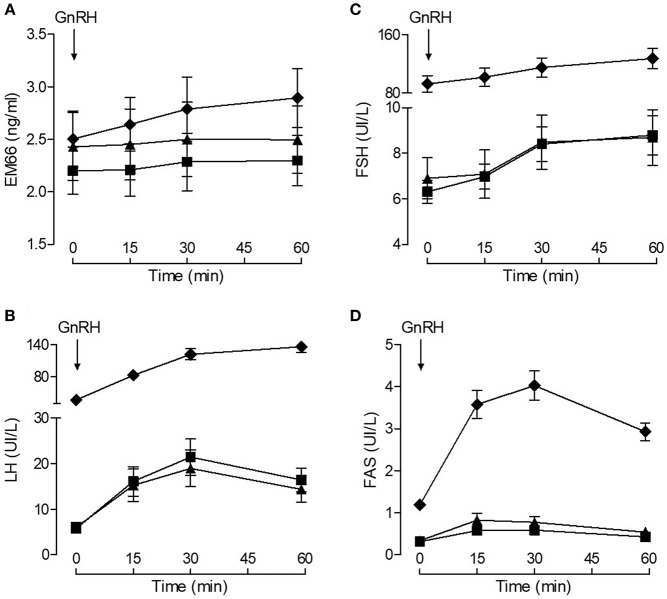
Plasma EM66 and gonadotropin levels of healthy women during stimulatory condition of the reproductive axis. Results of dynamic test for evaluation of EM66 **(A)**, LH **(B)**, FSH **(C)**, and FAS **(D)** levels in plasma of healthy women. Response of EM66 and gonadotropins after intravenous administration of 100 μg GnRH in premenopausal women without (■) or with (▴) oral contraception or in postmenopausal women (♦). Values are means ± SEM (*n* = 8).

**Figure 4:**
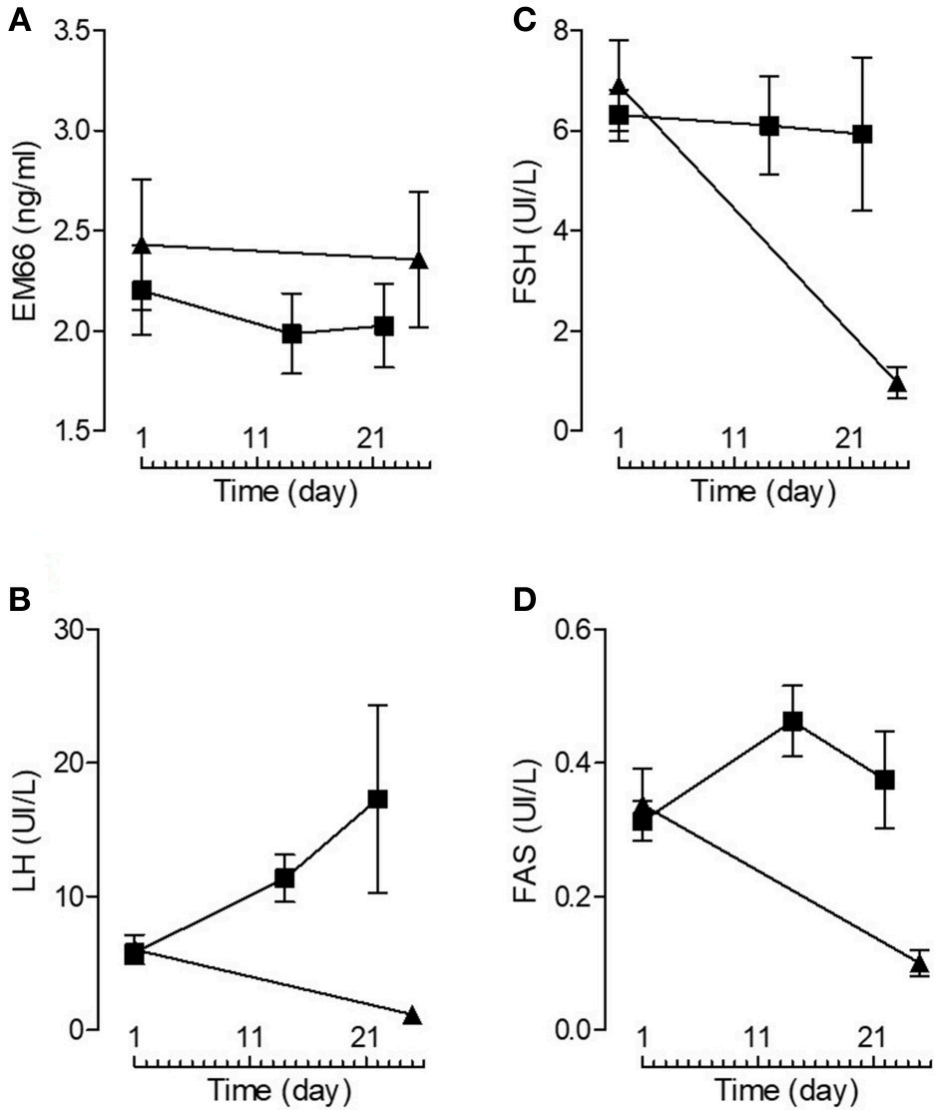
Plasma EM66 and gonadotropin levels of healthy women. Plasma EM66 **(A)**, LH **(B)**, FSH **(C)**, and FAS **(D)** concentrations during the normal menstrual cycle (women without oral contraception, ■) and in women with oral contraception (▴). Values are means ± SEM (*n* = 8).

Plasma EM66 concentrations did not change significantly after GnRH treatment in premenopausal women without or with oral contraception or in postmenopausal women (Figure 3A) in contrast to plasma LH (Figure 3B), FSH (Figure 3C), and FAS (Figure 3D). Moreover, on day 14 of the cycle (ovulation peak) and on day 22 (luteal phase), plasma EM66 levels were similar to those obtained at the day 1 of the cycle in women without oral contraception (Figure 4A). In women with contraception, plasma EM66 concentrations were similar between day 1 and day 25, which correspond to the few days of contraceptive treatment stop during the menstrual cycle and to 25 days of contraceptive treatment, respectively (Figure 4A). In contrast, plasma LH (Figure 4B), FSH (Figure 4C), and FAS (Figure 4D) levels were modulated during the menstrual cycle.

### Immunohistochemical Detection of EM66 in Pituitary Tumors

**Figure 5:**
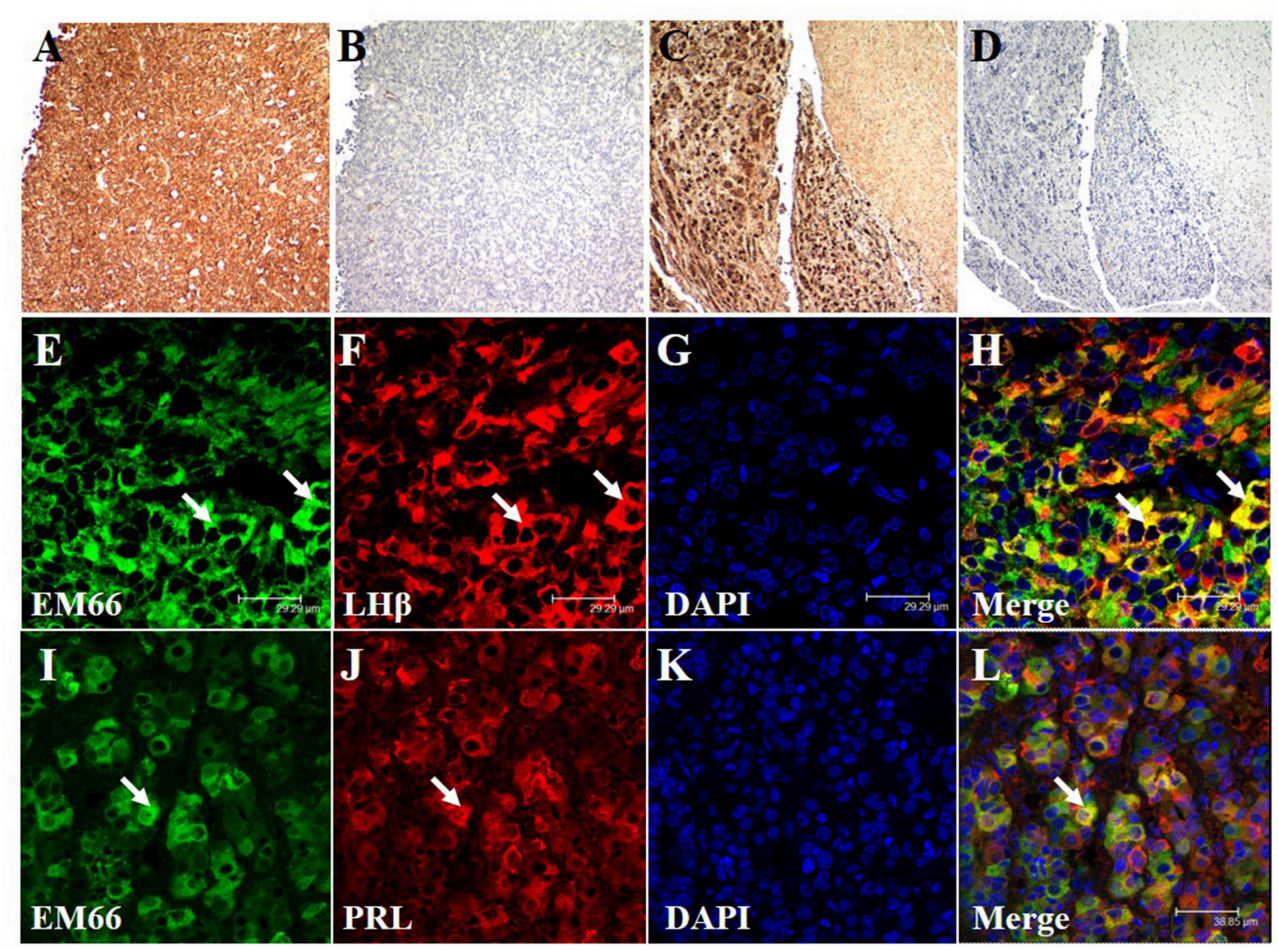
Immunohistochemical staining of EM66 and pituitary hormones in pituitary tumors. EM66-immunoenzymatic labeling of sections of a pituitary gonadotroph tumor **(A)** and a normal pituitary tissue **(C)** counterstained with hematoxylin. Substitution of the EM66 antiserum with nonimmune rabbit serum totally abolished the immunostaining in gonadotroph tumor **(B)** and in normal pituitary tissue **(D)**. EM66 **(E)**, LHß **(F)**, and DAPI **(G)** immunofluorescence staining were visualized in gonadotroph tumor section. Colocalization of EM66 and LHß is shown in the merged photomicrograph (**H**, *arrows*). EM66 **(I)**, PRL **(J)**, and DAPI **(K)** immunofluorescence staining were visualized in lactotroph tumor section. Colocalization of EM66 and PRL is shown in the merged photomicrograph (**L**, *arrow*). Scale bars, **(A–D)** 100, **(E–H)** 29.29, and **(I–L)** 38.85 μm.

Immunoenzymatic labeling of sections from a pituitary gonadotroph tumor (Figure 5A) and from a normal pituitary tissue (Figure 5C) produced intense EM66 staining of cells. When the EM66 antiserum was substituted with non-immune rabbit serum, no immunostaining was observed (Figures 5B,5D). Co-incubation of pituitary gonadotroph tumor slices with the anti-EM66 and the anti-LHß antibodies revealed that gonadotroph cells contain strong EM66 immunoreactivity (Figures 5E–H). However, LHß-EM66 colocalization is not observed in all cells since some LHß-positive cells did not exhibit any EM66 immunoreactivity and some EM66-positive cells did not display LHß immunoreactivity (Figures 5E–H), indicating that the peptide occurs in cells other than gonadotrophs. Co-incubation of pituitary lactotroph tumor slices with the anti-EM66 and PRL antisera showed that some EM66-positive cells were lactotrophs (Figures 5I–L). Finally, we noticed in the samples analyzed that >50% of cells are EM66 and LHß-positive in the cases of gonadotroph tumors. In contrast, only few PRL-positive cells were also EM66-positive.

### Characterization of EM66 Immunoreactivity in Pituitary Gonadotroph Tumor Extracts

**Figure 6:**
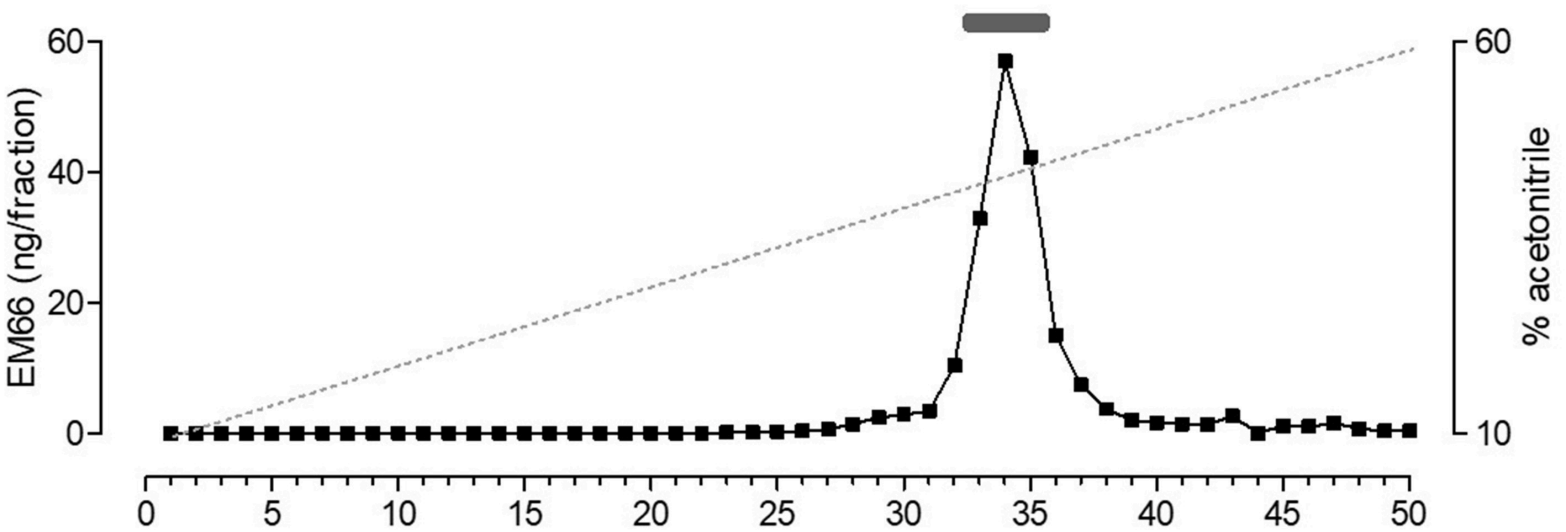
Reversed-phase HPLC analysis of EM66 immunoreactivity in pituitary gonadotroph tumor. The bar above the peak indicates the elution position of synthetic EM66 chromatographed the same day as the sample. The dashed line shows the concentration of acetonitrile in the eluting solvent.

Biochemical characterization of EM66 immunoreactivity in the pituitary gonadotroph tumors was performed by HPLC analysis combined with RIA quantification. Synthetic EM66 eluted in fractions 33–35 corresponding to a concentration of acetonitrile of 43–45% (Figure 6). A major immunoreactive peak eluting at the same position as synthetic EM66 was detected in extracts of gonadotroph tumors (Figure 6).

### EM66 Levels in the Plasma of Patients With Gonadotroph or Lactotroph Tumors

**Figure 7:**
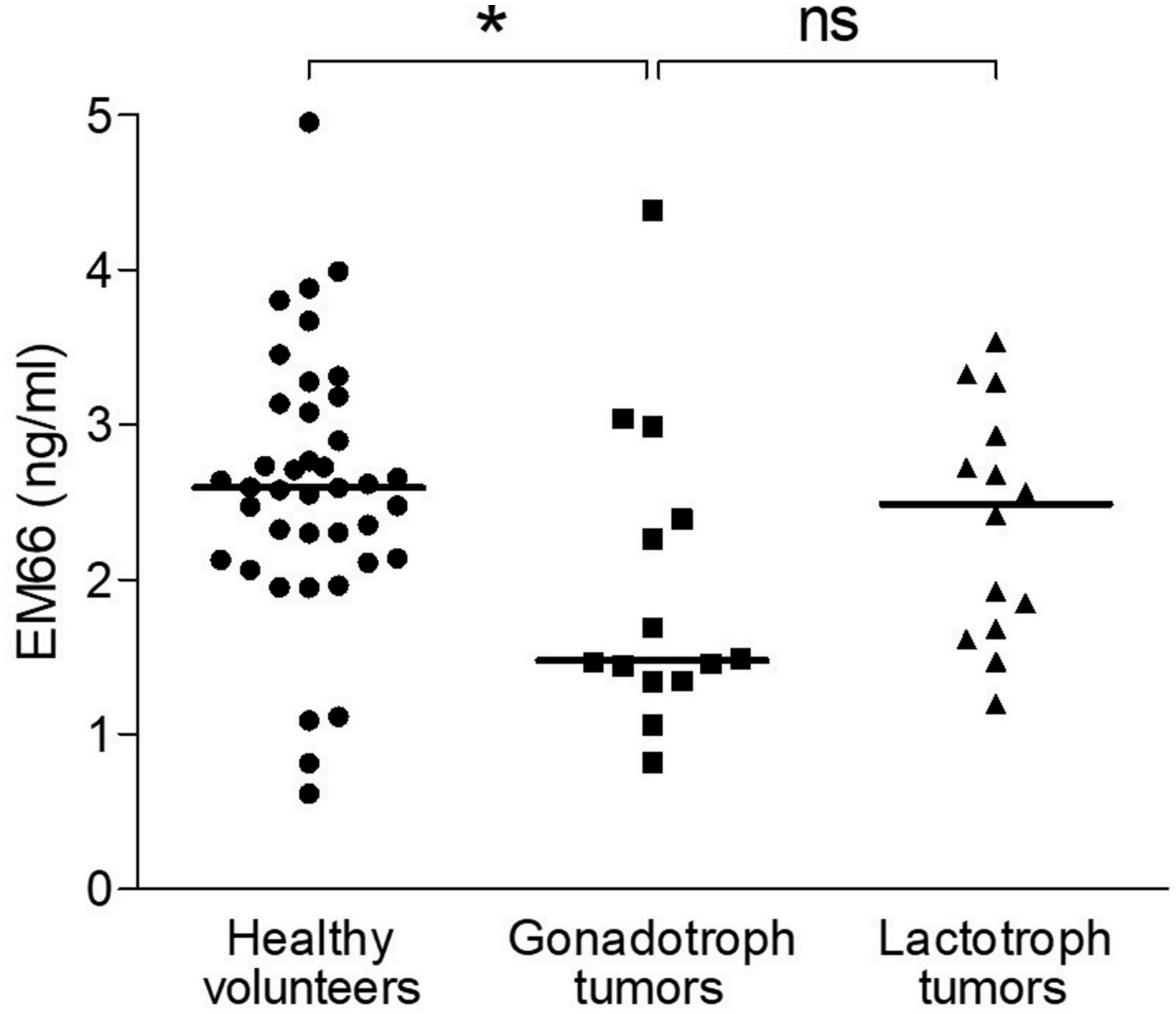
EM66 levels in plasma of patients with pituitary tumor. Scattergram of plasma EM66 concentrations in healthy volunteers (•, *n* = 40) and patients with gonadotroph tumor (■, *n* = 14) or lactotroph tumor (▴, *n* = 14). The bars represent the median value for each group. *p < 0.05; ns, non-significant.

Because data for healthy men and women were similar, they were pooled together for the following analysis. In this healthy volunteer group (*n* = 40), plasma EM66 concentrations ranged from 0.62 to 4.95 ng/ml with a median value of 2.59 ng/ml (Figure 7). The concentrations of EM66 in gonadotroph tumor patients (*n* = 14) ranged from 0.82 to 4.38 ng/ml with a median value of 1.48 ng/ml whereas the concentrations of EM66 in lactotroph tumor patients (*n* = 14) ranged from 1.19 to 3.54 ng/ml with a median value of 2.49 ng/ml (Figure 7). Statistical analysis revealed that the median value of EM66 concentrations was significantly lower (*p* < 0.05) in patients with gonadotroph tumor than in healthy volunteers (Figure 7). EM66 concentrations were not significantly different between patients with pituitary gonadotroph or lactotroph tumors (Figure 7) and for this last group, the EM66 median value was not significantly different compared to healthy volunteers (Figure 7). Moreover, the concentrations of EM66 in men and women were not significantly different in pituitary gonadotroph or lactotroph tumor patients (not shown). Finally, no correlation was found between plasma EM66 levels and plasma LH or FSH levels in gonadotroph tumor patients (not shown).

## Discussion

In this study, we evaluated within the framework of a clinical trial EM66 concentrations in plasma of healthy volunteers in different physiological conditions of the gonadotroph axis, we characterized the occurrence of EM66 in pituitary tumors and we also evaluated the plasma EM66 levels in a cohort of patients with gonadotroph or lactotroph tumor.

In our previous studies, comparison of EM66 levels in plasma of healthy volunteers revealed no gender differences (12). In the present study, we confirmed that basal EM66 levels are similar between men and women and we also extend this result to other groups of healthy volunteers i.e., premenopausal women with or without contraception, pregnant, and postmenopausal women. We have also demonstrated that plasma EM66 levels did not change during stimulatory or inhibitory conditions of the gonadotroph axis in men or women. It has been previously reported that SgII is a major product of gonadotrophs, in which its biosynthesis and secretion are regulated by GnRH and sex steroids (24–27). More precisely, SgII resides within secretory granules in gonadotrophs and is released along with LH after GnRH stimulation (26, 28). In addition, treatment of the mouse pituitary gonadotroph cell line LβT2 with GnRH increased SgII mRNA levels as well as secretoneurin (SN, an EM66 flanking-peptide that also derives from cleavage of SgII) levels in the culture media after static incubation (29). However, it was observed that pulsatile GnRH stimulation also increased the secretion of SN but did not alter SgII mRNA levels in LβT2 cells (30). Interestingly, we have previously observed that pituitary adenylate cyclase-activating polypeptide (PACAP) differentially regulates the secretion of EM66 and SN in primary bovine chromaffin cells (31, 32). Indeed, PACAP induces the release of pre-existing pools of SN after 5 min of treatment whereas the effect on EM66 secretion was observed only after 6 h of treatment and is dependent on SgII biosynthesis (3). Therefore, we can speculate that measurement of plasma EM66 levels 60 min after GnRH stimulation *in vivo* is not long enough to detect EM66 variation.

Gonadotroph tumors account for 25–35% of all pituitary tumors, constitute the majority of clinically non-functioning tumors and are thus lately diagnosed at the occurrence of signs and symptoms of tumor mass effects (33–35). The lack of specific plasma markers for these tumors results in a difficulty to diagnose these neoplasms early and to discriminate them from other pituitary tumors, thus emphasizing the crucial need to search new circulating markers for these tumors. SgII has been described as a major product of gonadotrophs (15) and it is highly expressed in human pituitary gonadotroph tumors (16) suggesting that SgII or its derived peptides may be useful for the diagnosis of gonadotroph tumors. Moreover, an intense EM66-immunoreactivity has been detected in gonadotroph cells of the rat pituitary (2). Together these observations suggest that this SgII-derived peptide may represent a sensitive circulating marker for the diagnosis of gonadotroph tumors. In the present study, we demonstrated that EM66 is produced in human gonadotroph tumors. Indeed, double labeling of a gonadotroph tumor with EM66 and LHβ antibodies revealed that EM66 immunoreactivity is localized to gonadotroph cells whereas HPLC analysis combined with RIA detection resolved a single immunoreactive peak co-eluting with synthetic EM66 in gonadotroph tumor extracts. Thus, the occurrence of EM66 in gonadotroph tumoral cells suggests that this peptide could also be released in the circulation of patients with gonadotroph tumor.

In other pituitary tumors, we have observed that EM66 is localized in few lactotroph and corticotroph cells of lactotroph and corticotroph tumors, respectively, whereas somatotrophs were totally devoid of EM66 labeling in somatotroph tumors (not shown). In agreement with this observation, a previous study has reported that SgII immunoreactivity was absent from somatotroph tumors and that only few lactotroph cells were SgII-immunopositive in human pituitary (16). Together, our data demonstrate that, in gonadotroph, lactotroph and corticotroph tumors, SgII serves as a precursor to generate the peptide EM66 as previously observed in pheochromocytomas (8). In addition, the weak immunoreactivity of SgII and EM66 in human lactotrophs may explain the absence of variation of plasma EM66 levels in patients with lactotroph tumor compared to healthy volunteers.

In patients with gonadotroph tumor, while the median value is statistically different, we have observed that plasma EM66 concentrations were in the same range as those of controls indicating that measurement of plasma EM66 is not a circulating marker for the presence of gonadotroph tumors and cannot constitute a diagnostic tool for these tumors. The majority of gonadotroph tumors are clinically non-functioning tumors i.e., do not secrete more hormone than normal pituitary (34, 36) which may explain why plasma EM66 levels are not increased in patients with gonadotroph tumors. Similarly, it has been demonstrated that these tumors may release low amounts of chromogranin A (CgA) without any influence on plasma CgA concentrations (37). In this context, measurement of plasma CgA, which is the best general plasma marker of neuroendocrine tumors (38) is not recommended for diagnosis of pituitary gonodotroph tumors (38, 39). Interestingly, while plasma EM66 concentrations in patients were in the same range as normal values, the median value of EM66 levels was significantly lower in the group of gonadotroph tumor patients than in the group of healthy volunteers. This observation suggests that EM66 is released into the circulation in smaller quantities by tumoral compared to normal gonadotroph cells. EM66 is a SgII-derived peptide generated through endoproteolytic cleavage by proprotein convertases PC1/3 and PC2 (10, 40, 41). Therefore, the lower plasma EM66 levels in patients with gonadotroph tumor could be related to a lower proteolytic activity of PC1/3 and PC2 in the tumors as already suggested for CgA processing in pituitary tumors (42, 43).

Finally, even if the majority of gonadotroph tumors are clinically non-functioning, some of them are functioning tumor with signs of hyperstimulation and/or increase of plasma gonadotropins (44). In these tumors, especially in women, 80% of the cells are strongly positive with anti-LH and FSH antibodies (45, 46). The plasma levels of gonadotropins are slightly increased and the tumoral hypersecretion is not diagnosed because it is considered as menopausal levels (44, 47). Thus, it will be very interesting to evaluate the plasma EM66 levels in such functional gonadotroph tumors.

In conclusion, our study allowed us to determine plasma EM66 concentrations in healthy volunteers in different physiological conditions in relation to gonadotroph status, and to show that these concentrations are independent of the stimulation or the inhibition of the gonadotroph axis. This study has also demonstrated that EM66 is present in gonadotroph, lactotroph, and corticotroph pituitary tumors but that plasma EM66 is not a useful marker for the diagnosis and the follow-up of gonadotroph or lactotroph tumors. Considering that EM66 is a sensitive marker for adrenal tumors, the measurement of this peptide in plasma of patients with other neuroendocrine tumors is needed to evaluate the specificity of this marker.

## Author Contributions

JG analyzed data, prepared figures, and wrote the manuscript. MG conducted the experiments. A-FC collected plasma and tissue samples and performed clinical diagnosis. A-GL collected data from patients. J-MK, YA, and LY designed the clinical trial protocols, and critically revised the manuscript. LY supervised the experiments. All authors approved the final manuscript.

### Conflict of Interest Statement

The authors declare that the research was conducted in the absence of any commercial or financial relationships that could be construed as a potential conflict of interest.
